# Host-Derived Proteomic Profiling of Human and Canine Mammary Tumor Xenografts Identifies Enrichment of Lipid Metabolism-Related Pathways

**DOI:** 10.1007/s12013-026-02111-2

**Published:** 2026-08-22

**Authors:** Elisa Díaz-Grijuela, Saioa Goñi, Alejandro Urdiciain, Miguel Barajas, Ileana B. Gonzalez-Valdes, Maider Espinal, Claudia Caballero-Mínguez, Enrique Santamaria, Agustín Hernández, Egoitz Astigarraga, Gabriel Barreda-Gómez, Raquel Urtasun, Joaquín Fernandez-Irigoyen

**Affiliations:** 1 Health Science Department, Public University of Navarre, Pamplona, 31006 Spain; 2 Betternostics SL, Noáin, 31110 Spain; 3 Proteomics Unit, Clinical Neuroproteomics Laboratory, Hospital Universitario de Navarra (HUN), Universidad Pública de Navarra (UPNA), Instituto de Investigación Sanitaria de Navarra (IdiSNA), Irunlarrea 3, Navarrabiomed, Pamplona, 31008 Spain

**Keywords:** Translational Oncology, Animal Model, Molecular Biomarker, Orthologous Proteins, Mass Spectrometry

## Abstract

Breast cancer is the most frequently diagnosed cancer in women worldwide and one of the most common malignancies in dogs. Notable clinical, hormonal and molecular similarities between both species suggest that dogs may serve as a valuable comparative oncology model. Alterations in lipid homeostasis and metabolism are closely associated with the acquisition of cancer hallmarks. In this study, a comparative proteomic analysis of murine host-derived proteins was performed in cell line-derived xenograft (CDX) models generated from human triple-negative breast cancer cell line (MDA-MB-231) and a canine mammary carcinoma cell line (CMT-U27). Using LC-MS/MS and species-resolved proteomics, tumor-associated changes in the host proteome were characterized across models. In total, 577 proteins were differentially identified in tumor tissues compared with healthy mammary gland samples in this interspecies CDX comparison, with 51.99% showing increased and 46.79% decreased relative abundance, while a small proportion displayed opposite regulation patterns between models. Functional enrichment analysis revealed that several biological processes were altered, including pathways related to lipid metabolism. Moreover, proteins involved in lipid pathways showed a moderate level of concordance between models (*r* > 0.6, Mann-Whitney U test *p* < 0.05). Overall, these findings provide a descriptive overview of host-derived proteomic alterations in interspecies mammary tumor CDX models and suggest that lipid metabolism-related pathways may be involved in tumor-associated microenvironmental changes. This study should be considered exploratory and hypothesis-generating, providing a basis for future studies incorporating orthogonal validation and controlled experimental designs.

**Supplementary Information:**

The online version contains supplementary material available at https://doi.org/10.1007/s12013-026-02111-2.

## Introduction

Breast cancer (BC) is the most common malignancy worldwide in humans, with an estimated 2.45 million new cases in 2025 [1]. According to the Global Cancer Observatory of the International Agency for Research on Cancer (IARC), BC presents age-standardized incidence and mortality rates of 46.8 and 12.7 per 100,000 women, respectively [2]. Triple-negative breast cancer (TNBC), defined by the absence of estrogen and progesterone receptor and HER2 expression, accounts for 10–20% of cases and is associated with poor prognosis due to its aggressive clinical behavior [3].

In veterinary medicine, canine mammary tumor (CMT) is also highly prevalent in bitches, representing 50–70% of all tumors [4–6], with incidence rates approximately 260 cases per 100,000 in unsterilized animals [7–9]. Approximately 50% of CMT are malignant [6], and TNBC prevalence varies widely between 14% and 76% of cases [10–12].

Human and canine BC share key clinical and molecular features, including hormonal influence, age and obesity as a risk factor, and genetic alteration such as mutations in *BRCA1*, *BRCA2*, *TP53* or *ATM* [12–17]. These similarities, together with the high genomic homology between species [18], support the use of canine models in comparative oncology. Indeed, comparative analyses have shown substantial overlap in gene expression patterns between human and canine tumors [19].

Cell line-derived xenograft (CDX) models are widely used in cancer research due to their reproducibility, experimental and well-characterized biology [20, 21]. However, tumor engraftment efficiency depends on tumor subtype, and TNBC models do not always achieve optimal implantation rates [20]. In this context, canine-derived CDX models represent a promising yet underexplored approach that may better reflect disease heterogeneity and translational relevance.

Comparative CDX models enable the study of conserved tumor-host interactions under identical in vivo conditions, providing a framework to evaluate shared molecular features across species. Notably, species-resolved proteomics allows discrimination between human, murine and canine proteins, enabling the characterization of host microenvironment remodeling.

Cancer cells exhibit metabolic reprogramming that enables the maintenance of several cancer hallmarks, such as proliferation, apoptosis avoidance, and invasion capability, among others. For this, energetic and biosynthetic metabolism must be adjusted, including lipid metabolism, which is crucial for membrane synthesis, cell signaling, and the production of bioactive molecules. In this context, the deregulation of proteins involved in lipid homeostasis (enzymes, transporters and metabolic pathway regulators) may contribute to meeting energetic and structural demands. Understanding these changes has provided new opportunities to identify potential diagnostic and prognostic biomarkers, as well as potential therapeutic targets.

Despite the widespread use of murine xenograft models in BC research, the extent to which tumor–host interactions are conserved across species remains insufficiently characterized at the molecular level. Mass spectrometry–based proteomics enables species-specific protein identification, allowing discrimination between human, mouse, and canine proteins within xenograft samples and facilitating cross-species analysis. We aimed to explore whether xenografts derived from human TNBC and CMT generate distinct yet partially overlapping host proteomic signatures, reflecting species-specific tumor biology superimposed on conserved oncogenic pathways. To test this, we established parallel CDX models using a well-established human TNBC cell line (MDA-MB-231) and a representative canine mammary carcinoma cell line (CMT-U27), selected based on its reported tumorigenic and invasive characteristics and its use as a comparative model of aggressive mammary tumors [22]. Comparative proteomic profiling of murine-derived proteins was performed to characterize shared and divergent tumor–host molecular adaptations in a translational framework. This approach provides an exploratory and descriptive proteomic landscape of host response and provides a basis for future hypotheses-driven studies and functional validation.

## Materials and Methods

### Cell Lines

The following mammary tumor cell lines were used: a human cell line derived from metastatic triple-negative breast adenocarcinoma, MDA-MB-231 (ATCC CRL-12532) and a canine cell line derived from invasive ductal mammary carcinoma, CMT-U27, kindly provided by Dr Ana Judith Perisé (Biomedical Research Unit, UIB-UAX, Madrid, Spain). Both models are characterized by aggressive and invasive phenotypes.

### Culture Conditions and Sample Preparation

All cells were cultured at 37 °C in a humidified atmosphere with 5% CO_2_. The MDA-MB-231 and CMT-U27 cell lines were maintained in RPMI-1640 medium (Gibco) supplemented with 10% fetal bovine serum (FBS) and 1% penicillin/streptomycin (P/S).

Prior xenograft implantation, cells were washed with PBS, trypsinized, and centrifuged at 1500 rpm for 5 min at room temperature. Approximately 2 million CMT-U27 cells (canine CDX) per mouse were resuspended in cold PBS, whereas 5 million MDA-MB-231 cells (human CDX) per mouse were resuspended in cold solution of Matrigel: PBS (1:1) prior to inoculation. Matrigel was used exclusively to facilitate MDA-MB-231 xenograft establishment and improve engraftment efficiency.

### CDX Model

This study included a total of 25 six-week-old female Athymic Nude-Foxn1nu mice (Charles River Laboratories). After a one-week acclimation period, MDA-MB-231 cells (5 × 10^6^) (*n* = 10) or CMT-U27 cells (2 × 10^6^) (*n* = 10) were inoculated subcutaneously into the flanks of athymic mice. As control group, mammary gland tissues from same age female athymic mice (*n* = 5) were used. All the animals were sacrificed by cervical dislocation once the tumor reached an approximate diameter of 1.5 cm or when predefined humane endpoint criteria were met, following institutional and internationally accepted guidelines for animal welfare. All the solid biopsies were snap- frozen and stored at -80 °C until analysis.

All procedures involving animals were approved by the Institutional Animal Care and Use Committee (IACUC) of the University of Navarre (CEEA) under the protocol CEEA/055 − 22 (approval date: 21/10/2022).

### LC-MS

Proteins were extracted from solid biopsies by homogenization in lysis buffer (8 M urea, 50mM DTT in 50 nM ammonium bicarbonate) followed by centrifugation at 14,000 x g for 1 h at 4 °C. Protein concentration was determined using the Bradford assay (Bio-Rad).

Fifty micrograms of protein were reduced with 20 mM DTT (30 min, 30 °C) and alkylated with 30 mM IAA (30 min, 30 °C, dark). Proteins were digested with trypsin (1:20, w/w) for 4 h at 37 °C, followed by overnight digestion (1:50, w/w). Peptides were purified using Oasis HLB 96-well µElution Plate (Waters), dried in a SpeedVac, and reconstituted in 0.1% formic acid (FA). Peptide concentration was measured using a NanoDrop™ spectrophotometer (ThermoFisher).

LC-MS/MS analysis was performed on an Evosep One system coupled to an Orbitrap Exploris 480 mass spectrometer (ThermoFisher Scientific). Peptides were separated on a C18 Aurora Elite column (75 μm x 15 cm, particle size of 1.7 μm; IonOpticks) using a 68 min gradient (Whisper Zoom method, 20 samples/day). Spray voltage was set to 1.9 kV and ion transfer tube temperature to 275 °C.

Data were acquired in DIA mode with a full MS scan (350–1000 m/z; resolution 120,000; max IT 50 ms; AGC 300%) followed by 25 MS/MS windows (20 Th isolation, 0.5 Th overlap; resolution 30,000; max IT 49 ms; AGC 1000%). Fragmentation was performed using HCD (30%). Data were recorded in profile (MS) and centroid (MS/MS) modes.

### Data Analysis

Dia data were processed using Spectronaut (v 17.3, Biognosys) by directDIA analysis (dDIA). MS1/MS2 calibration and tolerance were set to dynamic. Precursor charge was limited to 4, and fragment selection was intensity-based. Carbamidomethyl © was set as fixed modification, while oxidation (M), acetyl (protein N-term), deamidation (N), and Gln -> pyro-Glu were considered variable (maximum 3 per peptide). Trypsin specificity allowed up to two missed cleavages. Peptide and protein FDR were set to 1%, and only proteotypic peptides were used for quantification.

Human xenografts were searched against SwissProt-reviewed *Homo sapiens* (Feb 2024) and *Mus musculus* (Apr 2024) databases, while dog xenografts were searched against SwissProt/Trembl *Canis lupus familiaris* (Dec 2024) combined with the same mouse database. cRAP contaminants were included. Species-resolved protein identification was achieved by searching each xenograft dataset against a combined species-specific database (human + mouse for MDA-MB-231 CDX; dog + mouse for CMT-U27 CDX). Only proteotypic peptides — defined as peptides mapping uniquely to a single protein within the combined database — were used for protein quantification, thereby excluding peptides shared between the host (mouse) and the tumor species. Ambiguous protein groups identified solely through shared peptides were not reported as independent entries.

Reverse hits, contaminants, and proteins identified only by modified peptides were removed. Label-free intensities were logarithmized (base 2) and filtered (≥ 70% valid values in at least one group).

Statistical analyses were performed in RStudio (v2024.09.1) using Bioconductor packages [23–27]. Data were imputed (normal distribution) and normalized (width adjustment). Differential expressions were assessed using two-sample t-tests with Benjamini-Hochberg correction (FDR), while fold change (FC) estimation was performed using the limma package [26]. Results were filtered at p adjusted < 0.05 and ≥ 30% FC.

### Functional Analysis and Similarity Evaluation of Human and Canine Xenograft Tumors

Functional analysis was performed using KEGG database on KO (KEGG Orthology) identifiers associated with the detected proteins, with a particular emphasis on the “09103 Lipid metabolism” category.

Similarity between human and canine CDX tumors was evaluated by comparing pathway-level proteomic profiles following the approach described by Fang et al., [28], with adaptation. Pairwise Pearson correlation coefficients were calculated using fold-change (FC) values of proteins within each KEGG pathway. For each protein, the absolute difference in log-scale FC between models (Δabs) was calculated as a measure of divergence. Pathway-level similarity was assessed for each KEGG pathway with at least five proteins (*n* ≥ 5). The distribution of Δabs values within each pathway was compared with the background (all proteins) using the Mann-Whitney U test, testing for lower divergence within pathways.

## Results

### Cell Derived-Xenograft Model: Tumoral Growth

**Fig. 1:**
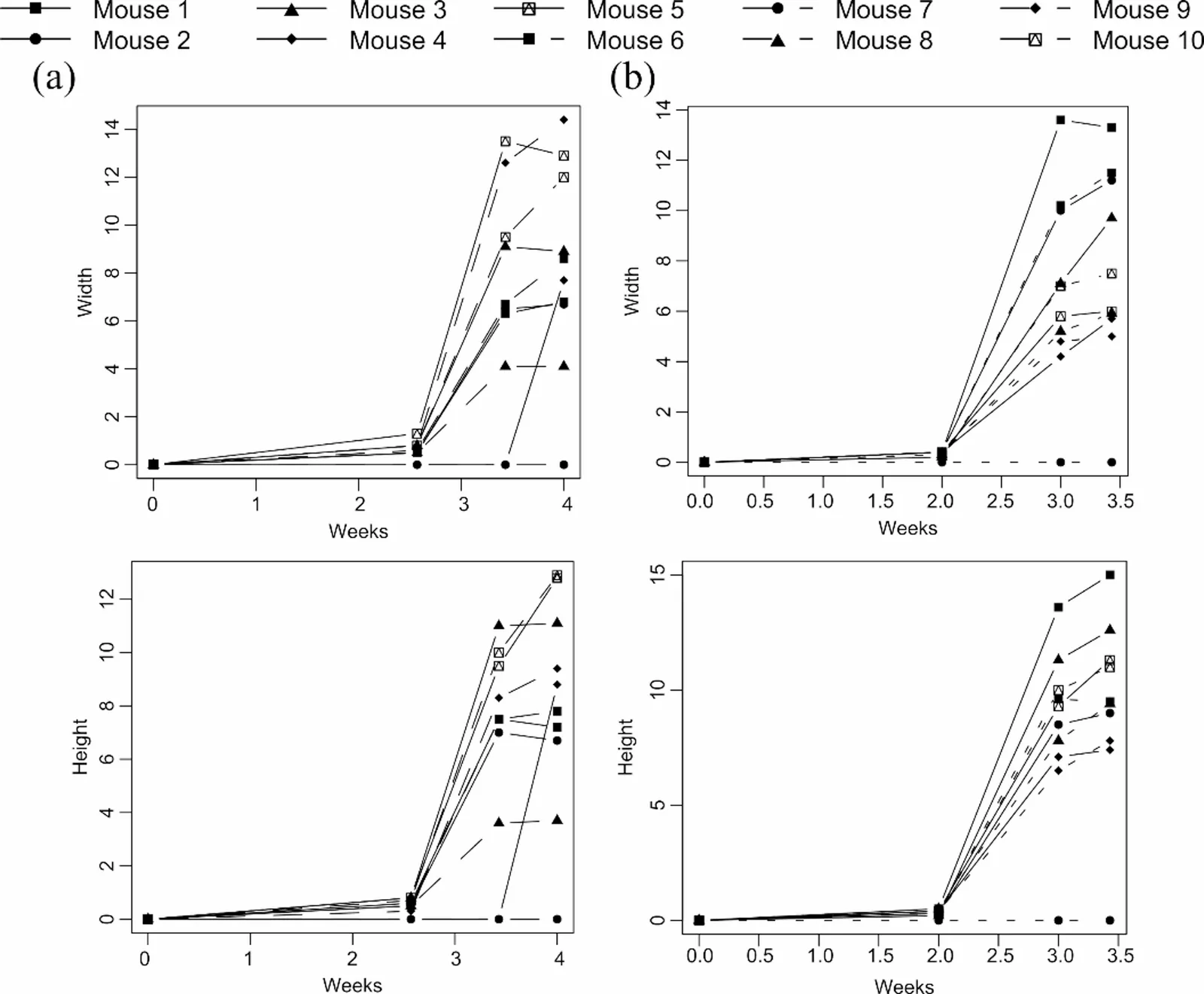
Assessment of individual tumor growth in interspecies CDX models of breast cancer. Representation of tumor dimensions (width and height, millimeters or mm) as a function of mouse age in weeks following inoculation (6 weeks, baseline or t0), corresponding to MDA-MB-231 cell line (**a**) and CMT-U27 cell line (**b**)

**Fig. 2:**
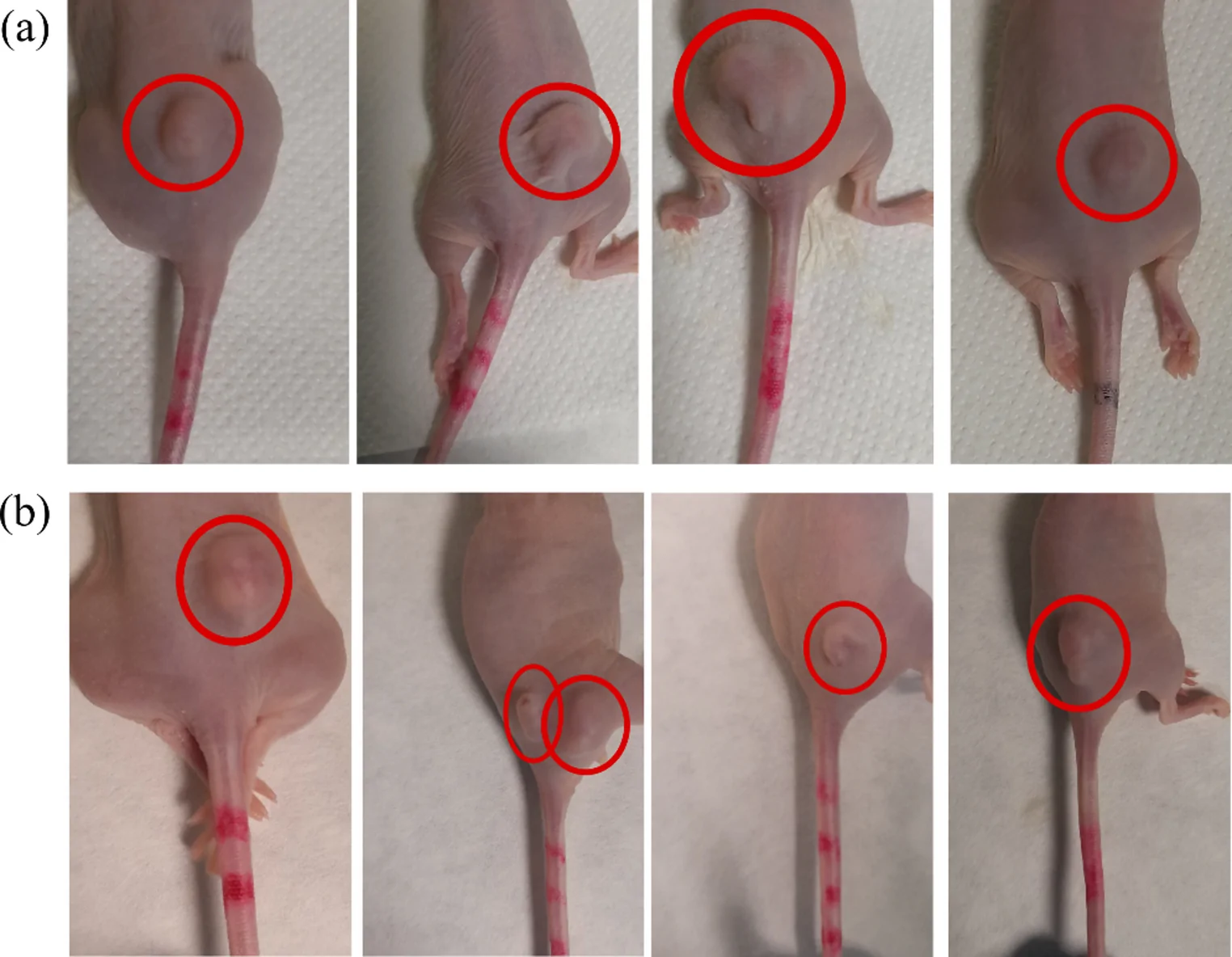
Interspecies cell-derived xenograft (CDX) model of breast cancer. Representative images of tumor growth at the endpoint of the study in CDX models derived from (**a**) MDA-MB-231 (from left to right: mice 2, 3, 5 and 6) and (**b**) CMT-U27 (from left to right: mice 2,3, 4 and 5)

Following inoculation with mammary tumor cell lines, athymic nude mice exhibited differential tumor growth depending on the number and species of origin of the cells used, reflecting differences in cellular aggressiveness under the conditions tested (Figs. 1 and 2). Tumor growth was additionally assessed by estimating tumor volume using the standard formula V = (width^2^ x height) /2. At the endpoint, mean tumor volume was 478.36 ± 400.48 mm^3^ and 445.13 ± 391.98 mm^3^ (mean ± sd) for MDA-MB-231 and CMT-U27 model respectively, considering only animals with detectable tumor growth.

Three weeks after inoculation, the MDA-MB-231 xenograft model began to show signs of tumor growth, with exponential growth observed one week later, reaching an appropriate tumor size after 4 weeks (Figs. 1a and 2a). Mouse 7 from the cohort was excluded for presenting a small mass suspected to correspond to Matrigel. Conversely, Mouse 4, despite showing no apparent tumor growth during the study, was found to harbor an internally growing tumor mass at sacrifice (Fig. 1a).

In the case of the CMT-U27 xenograft model, an adequate tumor size for the study was achieved four weeks before (Figs. 1b and 2b). In this case, Mouse 7 did not show any detectable tumor mass during the study; however, at the end of the study, the animal presented clinical signs suggestive of systemic deterioration, including marked swelling and reduced body temperature, raising suspicion of possible metastatic progression. Therefore, Mouse 7 was excluded from the study.

Despite both cell lines sharing the same breast cancer phenotype, the canine CDX model exhibited faster engraftment and growth under the tested conditions, requiring fewer cells and a shorter experimental timeframe.

### Proteomic-Based Similarity Between Interspecies CDX Tumors

Proteomic analysis of xenograft tumor tissues identified host-derived (*Mus musculus*) proteins associated with the tumor microenvironment in both mammary tumor CDX models. A total of 3124 and 2804 *Mus musculus -* derived proteins were identified from MDA-MB-231 and CMT-U27 xenograft tumor samples, respectively (Supplementary Table 1).

**Fig. 3:**
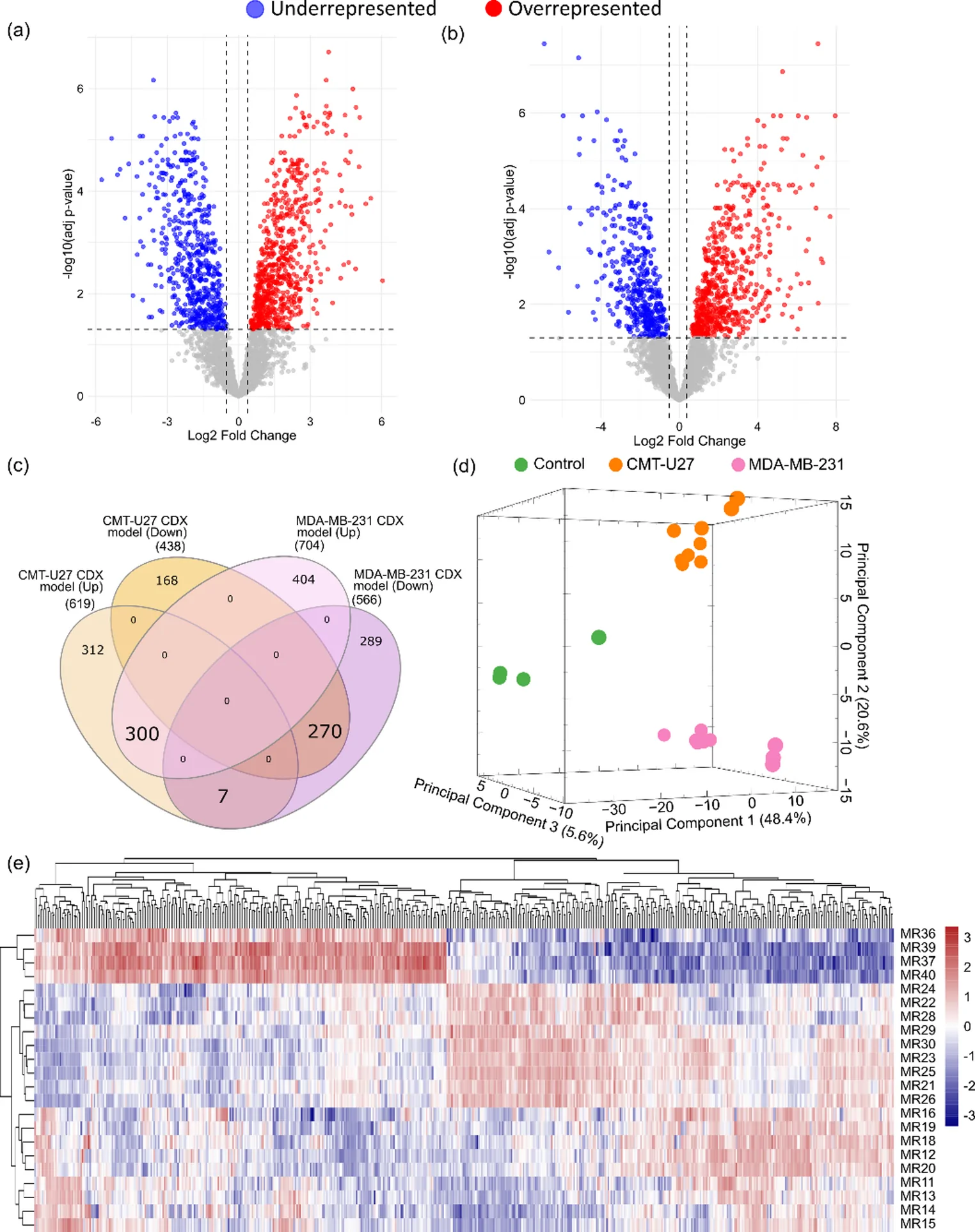
Statistical analysis of Mus musculus-derived proteins identified in solid biopsies from MDA-MB-231 and CMT-U27 CDX models. (**a**-**b**) Volcano plots representing changes in protein abundance (Log_2_ Fold Change) and statistical significance (-Log_10_ adj. p-value) in the (**a**) MDA-MB-231 and (**b**) CMT-U27 CDX models. (**c**) Venn diagram showing the overlap of differentially expressed proteins (adj p-value < 0.05) that are overrepresented (Up) or underrepresented (Down) in both CDX models. (**d**) Three-dimensional PCA of significant proteins common to CDX models. (**e**) Heatmap of significant proteins common to CDX models. MR36-MR40: control tissues (green), MR21-30: MDA-MB-231 CDX model (pink), MR11-20: CMT-U27 CDX model (orange), corresponding to mice 1–10 in each experimental group

The relative abundance of each protein was analyzed with respect to murine mammary tissues. This analysis identified 1270 and 1057 proteins with significant differences (adjusted p-value < 0.05) in the human and canine mammary tumor CDX models, respectively. Among them, 55.43% in the human model and 58.56% in the canine model were overexpressed in tumor tissues (Fig. 3a-b). Despite differences in the magnitude of proteomic alterations between human and canine CDX models, the comparative analysis revealed 300 overrepresented and 270 underrepresented proteins whose differential expression relative to healthy tissues was consistent between species. Only seven proteins showed an opposite FC direction (Fig. 3c). The low number of oppositely regulated common proteins supports directional concordance of the host response across xenograft species. These shared proteins were subsequently analyzed to identify enriched biological pathways associated with tumor microenvironment remodeling.

The three-dimensional PCA allowed the visualization of the distribution of solid biopsies samples based on their proteomic profiles (Fig. 3d). A clear separation was observed between healthy and tumor tissues along principal component 1 (PC1), reflecting differences in protein representation. Additionally, tumor tissues derived from the human and canine cell lines were differentially distributed along the second principal component (PC2). Therefore, proteins with higher loadings on PC1 are potential interspecies-conserved proteins associated with mammary tumor cell line and those with higher loadings on PC2 indicate proteins that are more species-specific (Supplementary Table 2). Consistent with these findings, heatmap-based hierarchical clustering showed a closer association between tumor samples from both species than with healthy tissue. Two opposing proteomic patterns were distinguished, indicating proteins selectively decreased or increased in tumors relative to normal tissue, with species-dependent gradient in expression levels (Fig. 3e).

**Fig. 4:**
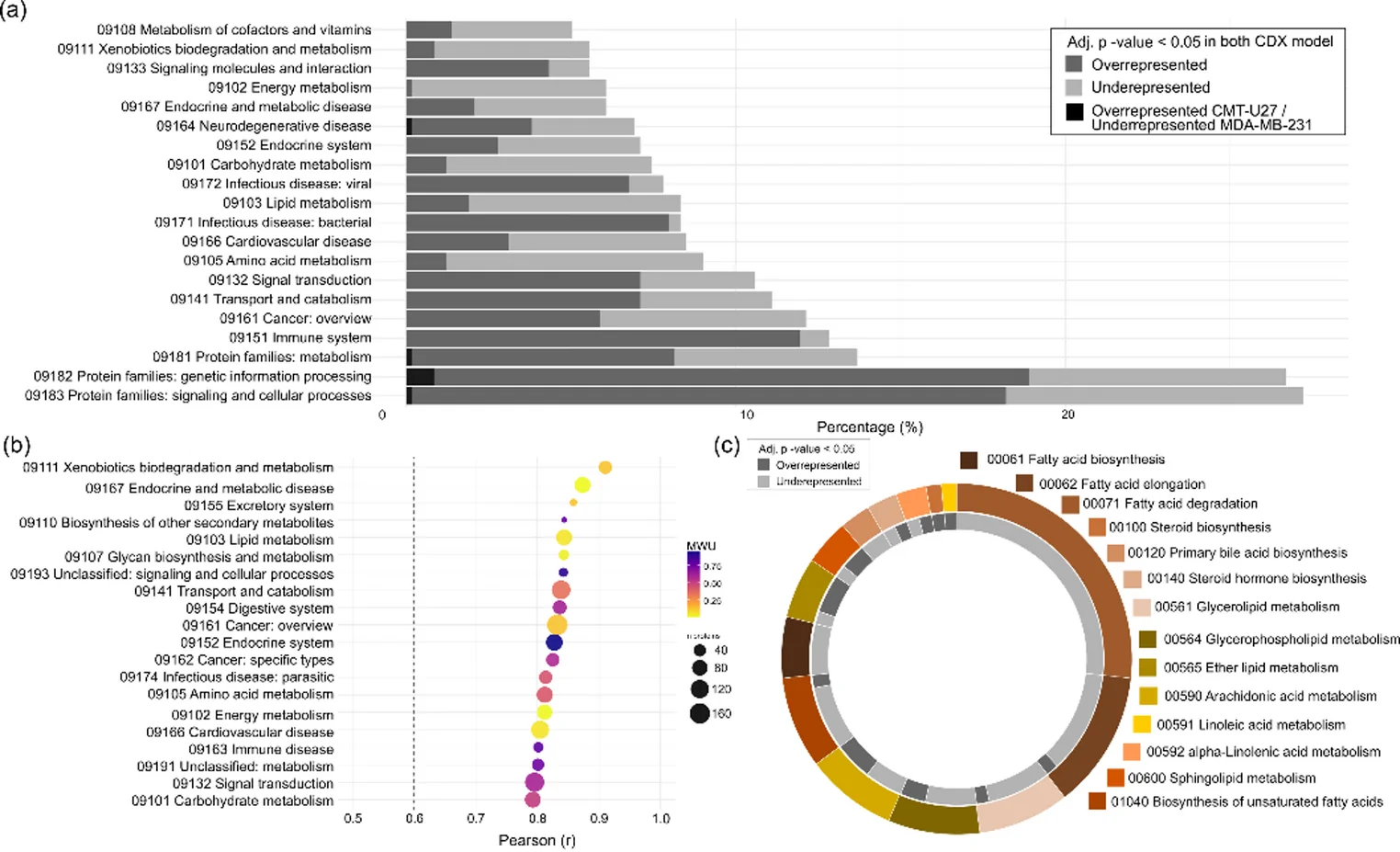
Functional analysis based on KEGG pathway annotations for Mus musculus-derived proteins, shared across the interspecies mammary tumor CDX model, identified in solid biopsy samples (577 proteins). (**a**) The 20 most abundant pathways, indicating up- or downregulated proteins across CDX models. (**b**) Top 20 KEGG pathway ranked by Pearson correlation showing similarity between CDX models (x-axis) and Mann-Whitney U test significance (colour); dot size represents the number of proteins. Pathway similarity was evaluated using proteins mapped to each KEGG category and compared against the global protein set (**c**) Circular plot of lipid metabolisms – related pathways, where the outer ring represents lipid metabolism pathways and the inner ring denotes regulation status (up/down) in both CDX models

Functional analysis based on the KEGG database, performed using the differentially significant proteins identified in both CDX models, enabled the identification of pathways with the highest number of altered proteins (Fig. 4a). These included transport-related pathways, signal transduction, amino acid metabolism and lipid metabolism, among others. For pathway-level similarity analysis (Fig. 4b), all those proteins differentially expressed and successfully mapped to KEGG pathways were considered, encompassing a total of 47 KEGG categories. Within this framework, lipid metabolism ranked within the top five pathways exhibiting the highest global similarity in regulation (*r* = 0.84). Furthermore, proteins associated with this process showed consistency between models compared with the global proteome (p MWU = 0.049). Taking together, these results suggest that proteins associated with lipid metabolism display relatively consistent patterns between models.

**Fig. 5:**
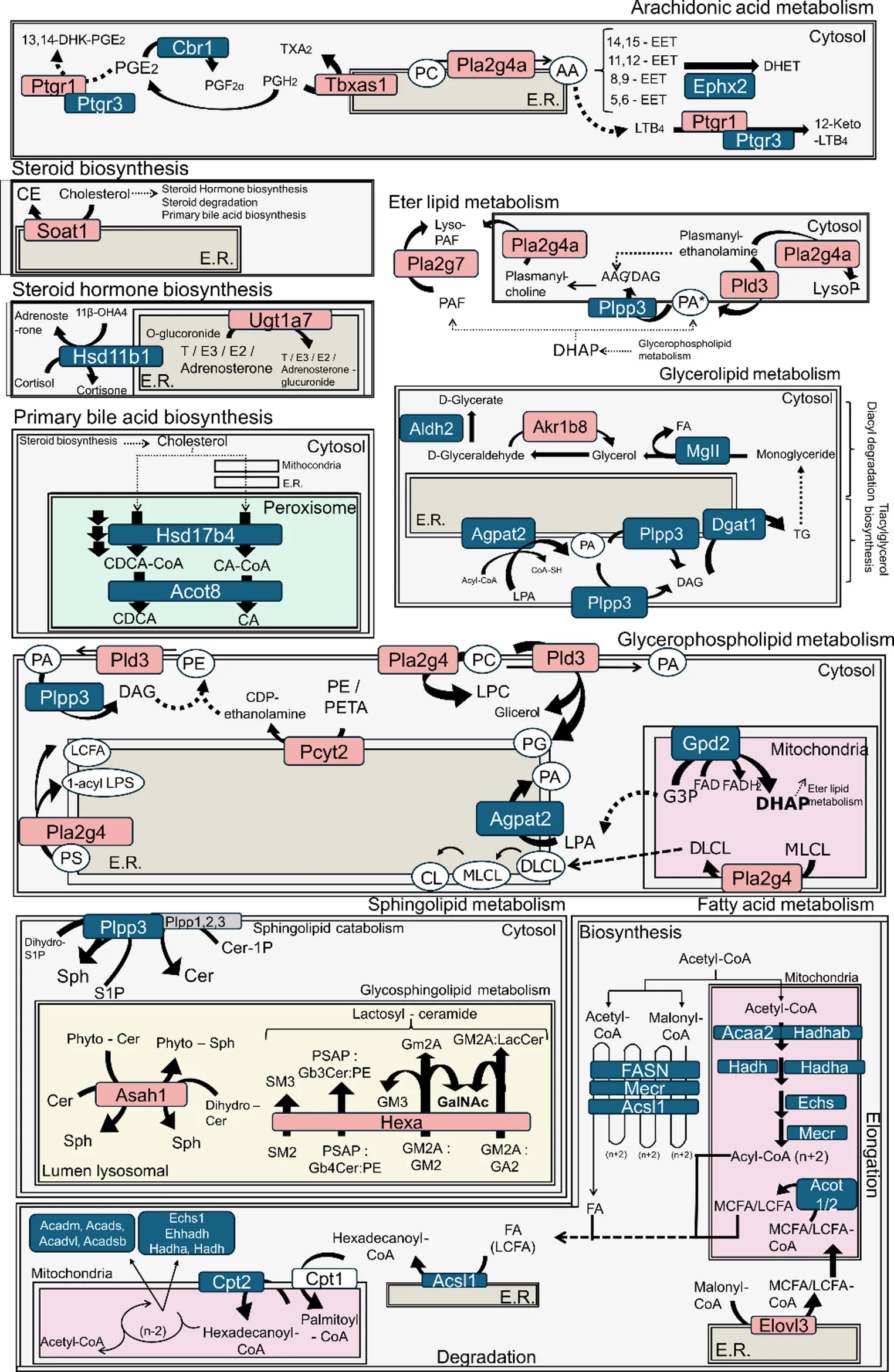
Schematic overview of lipid metabolism pathways, inspired by KEGG and Reactome maps, showing differentially identified proteins in both CDX models. Proteins boxed in red are overrepresented, whereas those boxed in blue are underrepresented in tumor tissues. Abbreviations: endoplasmatic reticulum (ER); DHK, dihydro-n-keto; PGE, prostaglandin E2; PGF, prostaglandin F; PGH, prostaglandin H; TXA, thromboxane A; LCFA, long-chain fatty acid; MCFA, medium-chain fatty acid; LPC/PC, glycerophosphocholines; LPA/PA*, glycerophosphates; PG, glycerophosphoglycerols; LPE/PE (or PETA), glycerophosphoethanolamines; LPS/PS, glycerophosphoserines; EET, epoxyeicosatrienoic acids; DHET: bioactive dihydroxyeicosatrienoic acids; LTB, Leukotriene B; CE, cholesterol ester; DHAP, glycerone-phosphate; PAF, 2-acetyl-1-alkyl-sn-glycero-3-phosphocholine; Lyso-PAF, 2-Acetyl-1-alkyl-sn-glycero-3-phosphocholine; AAG, 2-acyl-1-alkyl-sn-glycerol; DAG, di(acyl)glycerol; LysoP-, lysoplasmalogen; CDCA-CoA, chenodeoxylcholoyl-CoA; CDC, chenodeoxycholate; CA-CoA, choloyl-CoA; CA, cholate; OHA, hydrooxyandrost-4-ene-3,17-dione; T, testosterone; E2, estradiol; E3, estriol; TG, tri(acyl/alkyl)glycerol; FA, fatty acid; G3P, glycerol-3-phosphate; CDP, cytidine diphosphate; MLCL, monolysocardiolipin; DLCL, dilysocardiolipin; CL, cardiolipid; Cer, ceramide; S1P, sphingosine-1-phosphate; Sph, sphingosine; SM2, ganlgiotriaosylceramide-II3 sulfate; SM3, lactosyl-ceramide sulfate; GM, glucosylceramide; Gb, globoside; LacCer, lactosyl-ceramide; PSAP, prospaposine; GalNAc, N-acetylgalactosamine

Among the most representative pathways within lipid metabolism are fatty acid metabolism (biosynthesis, elongation and degradation), glycerolipid and glycerophospholipid metabolism (Fig. 4c). Among the most representative proteins in both CDX models, Fasn (MDA-MB-231 xenograft: logFC = -4.98, adj.p-value = 3.76E -5; CMT-U27 xenograft: logFC = -6.90, adj.p-value = 3.58E -8), Ehhadh (MDA-MB-231 xenograft: logFC = -3.80, adj.p-value = 2.73E -3; CMT-U27 xenograft: logFC = -4.94, adj.p-value = 1.15E -6), Acsl1 (MDA-MB-231 xenograft: logFC = -3.51, adj.p-value = 8.38E -5; CMT-U27 xenograft: logFC = -4.52, adj.p-value = 8.16E -3), Ephx2 (MDA-MB-231 xenograft: logFC = -3.65, adj.p-value = 4.52E-5; CMT-U27 xenograft: logFC = -6.90, adj.p-value = 3.58E -8) and Agpat2 (MDA-MB-231 xenograft: logFC = -3.25, adj.p-value = 4.27E-3; CMT-U27 xenograft: logFC = -3.02, adj.p-value = 3.09E -3) were found (Fig. 5).

In general terms, most pathways show a predominance of underrepresented proteins, indicating a downregulation of fatty-acid β-oxidation at both mitochondrial and peroxisomal levels. This effect is attributable to the underrepresentation of multiple short-, medium- and long-chain dehydrogenases (proteins of the Acad or Hadh families, Ehhadh, Echs1 and Cpt2), as well as thiolases (proteins of the ACA family) and enzymes associated with the oxidation of alcohols and aldehydes (Aldh2, Adh1 and Hsd11b1) (Fig. 5). Likewise, repression of de novo lipogenesis (fatty-acid biosynthesis and elongation) is observed, reflected by the underexpression of Fasn, Scd1, Acsl1 and Agpat2 (Fig. 5). Although FASN-driven lipogenesis is frequently described as a tumor-intrinsic vulnerability in TNBC, our data reflects the murine host compartment rather than the human/canine malignant cells themselves. Therefore, the observed downrepresentation of murine Fasn and related enzymes may indicate stromal/adipose remodeling and loss of normal mammary lipid-handling functions, rather than suppression of tumor-cell lipogenesis per se. This interpretation remains indirect and may also reflect differences in baseline tissue composition between normal mammary gland and tumor tissues. By contrast, the overrepresentation of Pla2g7, Pla2g4a, Tbxas1 y Ptgr1, involved in ether lipid and/or arachidonic acid metabolism – lead to an increased production of bioactive, inflammatory and enzymatically modified lipids (Fig. 5).

## Discussion

In this study, we investigated tumor microenvironment remodeling using a comparative xenograft approach involving human and canine mammary tumor cell lines. By specifically analyzing host-derived murine proteins, we were able to characterize stromal and metabolic responses induced by tumor growth independently of tumor-cell intrinsic proteomes. Mass-spectrometry-based proteomics has shown that implantation of tumor cells into the host animal leads to systematic alteration in the host proteome across host tissues and body fluids, reflecting changes in cellular processes, such as proliferation, apoptosis evasion and invasion, as well as the reprogramming of lipid metabolism, confirmed in this study, which may promote tumor growth [29]. Proteomic analysis of TME and host serum have identified host-derived proteins whose expressions change in response to tumor growth, supporting their potential use as diagnostic and prognostic biomarkers in cancer research [29].

The dog has become established as a valuable translational model in mammary tumors research due to the biological, clinical and molecular similarities it shares with BC [4–6]. Proteomic studies in in vitro models can help to identify common biomarkers and conserved molecular pathways between species, promoting a One Health approach in comparative oncology. The use of this model not only benefits veterinary medicine but also accelerates the development of diagnostic and therapeutic strategies applicable to human medicine. Therefore, the identification of differentially expressed proteins related to tumoral environment in CDX models that are conserved across species, may represent a key first step towards the development of biomarkers both for diagnosis and disease monitoring purposes.

In this study we investigated the host proteome of solid biopsies in breast cancer interspecies CDX models, specifically using a human TNBC cell line (MDA-MB-231) and a canine mammary carcinoma cell line (CMT-U27). To evaluate conserved proteins that were differentially expressed in solid biopsies, we compared *Mus musculus*-derived proteins from tumoral tissues with mouse mammary gland tissues. It should be noted that although Matrigel was used to facilitate MDA-MB-231 tumor establishment, previous studies have shown that, while it supports initial tumor take, it exerts limited influence on tumor growth kinetics and tumor microenvironmental features once tumors are established [30, 31]. The Student’s t-test with FDR adjustment identified 577 proteins with adjusted p-value below 0.05 and a minimum FC of 30% in both CDX models, which represent the 18.47% and 20.58% of the identified murine proteome in human and canine CDX models, respectively. Only 7 out of the 577 commonly differentially expressed proteins exhibited a FC in the opposite direction; by contrast, 300 proteins were identified as overrepresented and 270 as underrepresented in both models.

The functionality of this set of proteins was determined using KEGG database, to analyze which metabolic pathways or other relevant cancer-related functions may be altered in both species. Some of the enriched functions/processes included transport and catabolism, signal transduction and the immune system, with a higher proportion of overrepresented proteins in tumor tissues. In contrast, energy, amino acid, carbohydrate and lipid metabolism were mainly altered through negative regulation of the modified proteins. Nevertheless, lipid metabolism showed a high correlation between TME-associated changes in the CDX models, independently of the species of origin. In addition, lipid metabolism exhibited significant consistency at the proteomic level, as evidenced by significantly smaller differences between the two models compared with the overall set of identified proteins. In other words, proteins involved in this metabolic process display the same regulatory pattern with similar magnitudes and therefore behave in a conserved manner across species.

Regarding lipid metabolism, in both CDX models, proteins with higher FC were involved in FA metabolism pathways – Fasn and Acsl1 in FA biosynthesis, Echs1 in FA elongation, Hadh in FA elongation and/or degradation and Ehhadh in FA degradation -. Negative regulation within the TME showed a certain degree of consistency, considering that murine subcutaneous tissue is rich in adipocytes. This supports the possibility that, according to the literature, as the tumor grows, these adipocytes may dedifferentiate into cancer-associated adipocytes (CAA) [29, 32], thereby losing their FA oxidative capacity and lipogenic function. Likewise, the decrease in proteins such as Ehhadh may indicate a negative regulation of the stroma-dependent PPAR-α program [32], promoting a transition towards a more inflammatory and stressed TME, along with a reduced capacity to handle complex lipids [32, 33]. These alterations may reflect the replacement of normal murine adipose tissue by activated tumor stroma.

In the context of FA metabolism, which shows a generalized negative regulation, an increase in Elovl3 was identified. Elovl3 is involved in the elongation of long-chain saturated and monounsaturated fatty acids. This observation is consistent with the notion that lipid remodeling and adaptation within the TME are being promoted, favoring the incorporation of long-chain fatty acid (LCFA) into membranes and/or the generation of lipids involved in cell signaling [34, 35].

Another pathway involved is arachidonic acid metabolism, in which a significant negative regulation of Ephx2 was identified. Ephx2 is a key enzyme responsible for regulating arachidonic acid epoxides (EETs) by converting them into DHETs. The reduction in Ephx2 suggests an accumulation of EETs, which promotes angiogenesis and, consequently, enhances cellular proliferation and migration [36, 37]. Elevated levels of EETs in breast cancer tissues have been associated with increased tumor aggressiveness [38]. Moreover, high EET levels have been linked to the positive regulation of signaling pathways such as the PI3K/Akt and MAPK pathways [39, 40].

On the other hand, another protein showing a marked change in the murine microenvironment following xenograft tumor cell inoculation was Agpat2, which is involved in glycerophospholipid metabolism. Agpat2 catalyzes the conversion of lysophosphatidic acid (LPA) into phosphatidic acid (PA), a precursor of other phospholipids such as phosphatidylcholine (PC), phosphatidylethanolamine (PE) and phosphatidylinositol (PI) among others. In addition, PA functions as a signaling lipid involved in pathways such as mTOR and RAF and plays a role in lipid storage and membrane biogenesis. Within the context of the murine TME, Agpat2 decrease may be associated with a loss of functional adipocytes, consistent with the previously discussed transition towards CAAs [41, 42].

Several limitations should be considered when interpreting these results. First, xenograft models using immunodeficient mice do not fully recapitulate the complexity of immune interactions present in spontaneous tumors. Second, the comparison between xenograft tissue and healthy mammary tissue may introduce baseline differences in tissue composition that are independent of tumor-induced remodeling. Third, only one cell line per species was included, which limits the generalizability of the findings. Future studies incorporating multiple cell lines would be necessary to determine the extent to which the observed host-derived proteomic responses are consistent across different models of aggressive mammary tumors. Finally, additional validation using orthogonal experimental approaches would be required to strengthen the biological interpretation of the identified proteomic signatures.

Despite these limitations, our findings provide evidence that tumor growth induces conserved host metabolic responses across xenografts derived from different species. This supports the relevance of comparative oncology approaches for studying tumor–host interactions and identifying conserved biological processes associated with tumor progression.

In summary, the inoculation of human and canine mammary tumor cells induces a shift in the metabolic state of the murine TME, promoting lipid remodeling as well as the generation or preservation of bioactive lipids (EETs and/or LCFAs), and, consequently, promotes angiogenesis, inflammation and tumor support rather than lipid degradation. This comparative strategy captures conserved host-stromal responses under controlled in vivo conditions, although the use of athymic mice limits the interpretation of adaptive immune components of the TME.

## Conclusion

This study characterizes a host-derived proteomic response induced by human and canine mammary tumor xenografts. Comparative analysis suggests that lipid metabolism-related pathways may represent one of the most consistent biological signatures within the murine tumor microenvironment across both models. These findings support the value of comparative oncology approaches and reinforce the relevance of canine models for translational breast cancer research. Overall, this work provides a descriptive, hypothesis-generating proteomic framework that warrants further validation using orthogonal experimental approaches (e.g., transcriptomic or protein-level assays).

## Supplementary Information

Below is the link to the electronic supplementary material.

Supplementary Material 1

Supplementary Material 2

## Data Availability

The proteomic data obtained and/or analyzed during this study are available in the Supplementary Tables provided with the manuscript.
